# Reply to Chopra and Rizvi

**DOI:** 10.1093/cid/ciz128

**Published:** 2019-04-04

**Authors:** Sumanth Gandra, Anita Arora, Ramanan Laxminarayan, Eili Y Klein

**Affiliations:** 1 Center for Disease Dynamics, Economics & Policy, Washington, DC; 2 Fortis Healthcare Ltd., Gurgaon, Haryana, India; 3 Princeton Environmental Institute, Princeton University, New Jersey; 4 Department of Global Health, University of Washington, Seattle; 5 Department of Emergency Medicine, Johns Hopkins School of Medicine, Baltimore, Maryland; 6 Department of Epidemiology, Johns Hopkins Bloomberg School of Public Health, Baltimore, Maryland

To the Editor—Rising rates of antimicrobial resistance (AMR) are a global public health crisis that threatens to abrogate gains made in improving healthcare outcomes over the last 60 years. As our report [1] on more than 5000 patients in India makes clear, Gram-negative infections are significantly associated with mortality, and these infections are spreading rapidly around the globe in healthcare facilities as well as in the community [2]. Because our study was conducted in a private hospital setting and the vast majority of patients in India receive care in government hospitals that typically have less advanced care options, our result that patients with multidrug resistant Gram-negative infections were associated with 2–3 times higher mortality may actually understate the problem in India, as Chopra et al note [3]. Therefore, we echo the call that more data from government hospitals in India are urgently needed to better understand the burden of AMR in India. 

We believe that the Antimicrobial Resistance Surveillance Network initiated by the Indian Council of Medical Research [4], which includes both public and private hospitals, is an excellent start at complying with the Indian National Action Plan on AMR [5]. However, increased investment to strengthen laboratory capacity is urgently needed in many public and private hospitals to both improve surveillance as well as patient outcomes. Enhancements in surveillance will allow for better understanding of how disease severity and comorbid conditions impact mortality outcomes in patients with AMR infections. For instance, the contrast in mortality risk between our findings on non–intensive care unit patients [6] may be due to differences in infection location and disease severity. Overall mortality in the study of 116 patients by Naim et al. was only 4.3% (in contrast to 13.1% in our study), and none of their patients had bacteremia or lower respiratory tract infections (nearly 90% were from wounds or urine). In our study, the odds of mortality were significantly higher among patients with lower respiratory tract and bloodstream infections when compared with urinary tract and wound infections. Understanding these differences was only possible because of high data availability. Yet, national data are needed to understand the frequency of these infections and to develop national-scale plans to combat the problem of resistance.

While the scale of the problem is national and even global in nature, the outcomes are local. Thus, we further endorse the call by Chopra et al that there needs to be stringent implementation of antimicrobial stewardship and infection control activities to improve patient outcomes; however, greater investments are necessary on a country-wide level [7]. Large investments in training are needed as there are few infectious diseases physicians in India. These investments would likely bring significant returns as interventions by infectious diseases physicians in the United States are associated with improved outcomes and lower costs [8]. Furthermore, because resistance can spread rapidly around the world, greater investments are needed in AMR hot spots, such as India, to help contain and reduce the spread of resistance. In addition, increased investments in novel drug discovery is of significant importance as currently available therapeutic options are not effective against the common resistance mechanisms encountered in extremely drug-resistant Gram-negative bacteria in India.

## Notes

***Financial support.*** This work was supported by the Bill & Melinda Gates Foundation to the Global Antimicrobial Resistance Repository (grant OPP1112355) and the Global Antibiotic Resistance Partnership (grant OPP1135911) to the Center for Disease Dynamics, Economics & Policy as well as the Centers for Disease Control (grant 16IPA1609427) at Princeton University.

***Potential conflicts of interest.*** All authors: No reported conflicts. All authors have submitted the ICMJE Form for Disclosure of Potential Conflicts of Interest. Conflicts that the editors consider relevant to the content of the manuscript have been disclosed.
